# Perspectives of four stakeholder groups about the participation of female forest landowners in forest management in Georgia, United States

**DOI:** 10.1371/journal.pone.0256654

**Published:** 2021-08-24

**Authors:** Jacqueline Miner, Puneet Dwivedi, Robert Izlar, Danielle Atkins, Parag Kadam

**Affiliations:** 1 Warnell School of Forestry and Natural Resources, University of Georgia, Athens, GA, United States of America; 2 Land & Ladies, Brunswick, GA, United States of America; Shahjalal University of Science and Technology, BANGLADESH

## Abstract

As the number of female forest landowners (FFLs) in the United States continues to rise, there is an increasing need to understand the perceptions of stakeholder groups about opportunities and challenges faced by FFLs in the context of sustainable forestland management. This study utilizes the technique of SWOT-AHP (Strengths, Weaknesses, Opportunities, and Treats—Analytical Hierarchy Process) to understand the perceptions of four stakeholder groups (FFLs, private foresters, government representatives, and non-profits) in Georgia–a significant forestry state located in the Southern United States. Sixteen factors (four under each SWOT category) were selected through a comprehensive literature review and detailed interviews with individuals from the identified stakeholder groups. A survey was created using these factors that asked stakeholders to compare them in their respective SWOT categories. An additional survey was created for each stakeholder group where survey participants compared the highest-ranking factors in each SWOT category. We found that all stakeholder groups prioritized weaknesses over the other SWOT categories. Results showed a significant need for relevant educational outreach programs that cater specifically to FFLs. Additionally, researchers found a need to promote the interest of future generations in forestland management as all stakeholder groups felt that limited interest from future generations was the most important threat. This study will directly feed into regional, national, and international attempts to increase the participation of minority family forest landowners in sustainable forest management through integrated forest policy development.

## 1) Introduction

**Fig 1:**
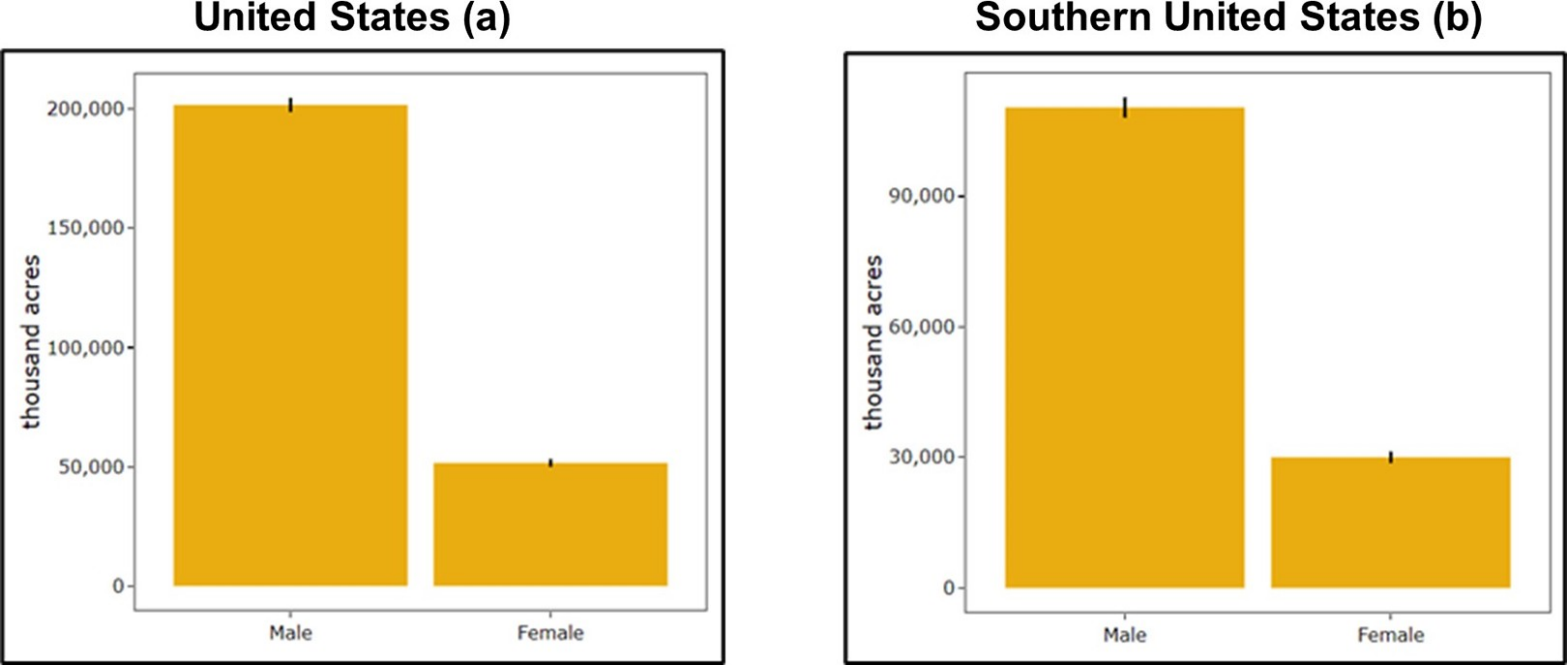
Gender representation in privately owned forestland [2].

The number of female forest landowners (FFLs) in the United States is increasing. Between 2006 and 2013, the percentage of FFLs in the United States who owned more than 10 acres of forestland rose from 12% to 14%, respectively [1]. The rise in the number of FFLs continued into 2018 when it was reported that 20.4% of all private forest landowners were female and owned about 50 million acres of forestland nationwide (Fig 1A, Butler et al., 2020a). A similar trend is also observed in the Southern United States. In 2013, 387,000 FFLs owned about 25 million acres of timberland in the region [1], meaning that approximately 17% of total timberland in the Southern United States was owned by FFLs [1]. Further, Butler et al. [2] found that in 2018, female private forest landowners owned about 30 million acres of forestland, equating to about 21.5% of total forestland in the Southern United States (Fig 1B). This increase in female forestland ownership throughout the country can be primarily attributed to the differences in life expectancy between men and women, where women are expected to live 5.4 years longer than men [3].

FFLs are typically less engaged in forest management than male forest landowners [4]. This could be attributed to the fact that forestry has historically been a male-dominated field, making it difficult for FFLs to start a conversation about forest management [5]. Additionally, forestry training manuals mainly cater to the male clientele, failing to create a sense of community among FFLs [6]. Finally, no substantial research efforts have been made to understand motivations and constraints faced by FFLs in the context of sustainable forestry at the national and regional levels. To the best of our knowledge, only Butler et al. [7] and Majumdar et al. [8] have evaluated the differences between male forest landowners and FFLs at the national level in the United States. Butler et al. [7] found that FFLs were more likely to have inherited land, particularly from a spouse and male forest landowners were more likely to actively manage their land for wildlife, have a commercial timber harvest, and have undertaken management activities in the last five years compared to FFLs. Additionally, Majumdar et al. [8] reported that FFLs are more likely to manage their forestlands for aesthetics and biodiversity than timber production.

A need exists to understand such differences at the regional level as motivations and constraints of FFLs could differ significantly across regions with different local cultural, societal, and economic norms [9]. This lack of knowledge about FFLs at the regional level has resulted in a situation where extension efforts to engage FFLs actively were either not launched or have failed due to a mismatch between information provided and information needed, resulting in lower recruitment and funding support [6]. Additionally, the forestry community has failed to create a peer-to-peer networking platform for FFLs that would have helped them share their experiences and provided a mutual learning opportunity for sustainable forest management [6]. This has led to the situation that male forest landowners are less likely to give away wooded land in the next five years by 6.5% relative to FFLs [10].

FFLs will ultimately be making critical decisions about the future of the forestlands in the United States, in general, and in the Southern United States, in particular. These decisions about possible actions regarding forestland (e.g., sold, bequeathed, subdivided, conservation easements, alternative land use, etc.) will determine the fate of forestry across the southern states. In this context, it is imperative to characterize FFLs located in the Southern United States first and then understand their intrinsic motivations and extension needs in the context of forest management. This will help to engage them in forest stewardship constructively. This will also ensure the sustainable management of forest resources in the Southern United States, thereby securing the position of the region as the wood basket of the world while providing forest-based ecosystem services for the growing population in the region, ensuring the prosperity in rural parts of the region, and facilitating the successful intergenerational land and knowledge transfer.

## 2) Goal and objectives

The goal of this study is to promote forest stewardship among FFLs for ensuring the sustainability of forest resources in the Southern United States. The objectives of the study are to understand the perspective of stakeholder groups about the factors affecting the participation of FFLs in forestry management and then to prioritize these factors for addressing the same in regional and national policy-level deliberations.

## 3) Literature review

The inclusion of gender-based perspectives in forestry activities can affect how practices are implemented by groups. Geographical variation of natural resources and gender-based demographic capital has been argued to be associated with the efficiency of environmental policies and programs as evidenced by the overlap of gender norms and taboos with specific spatial patterns of forest use [11]. Gender is one type of demographic difference whose social norms have created spaces for women’s invisibility in natural resources management. This is certainly true for the cases where explicit operationalization of ‘who counts’ is lacking during community consultation–in the social groups where there are invisible (or rather embodied) actors and their agencies [12]. This invisibility is historically constructed and culturally facilitated through the societal nature of the division of labor [13–15]. This division of labor between men and women about what work is expected from them, as well as with what capacity and agency, is the primary driver of roles and perceptions of men and women in the management of natural resources [16]. Knowledge in general and traditional knowledge specifically has gendered aspects of growth partially based on the division of labor in informal education and partially on cognitive differences between men and women [17]. Explicit inclusion of gendered governance discourse in natural resource management affects the use of such knowledge towards forestry [18, 19].

The forestry sector worldwide is embedded in the locally occurring cultural norms of who can own land, who has access to land, who can grow trees, and who can benefit from them. The normatively designated gender structures and roles for these four processes are irrevocably entangled with economic, religious, and racial ones. Norms of what kind of different customary rights women have and how women use public spaces differently have been argued to affect gender consideration in the efficiency of forestry programs [20]. Fortmann [21] notes that narratives about natural resource property rights and the nature of affected social networks create a social process that also affects gender considerations. The dialectic between norms and laws for gender consideration is important as it demonstrates the pathways in which gender biases solidify into accepted rules for natural resource management. Moreover, livelihood and commercialization of certain natural resources are also affected by the explicit exclusion of gender considerations. The evidence in the context of commercialization of natural resources is complicated as market-based conservation has been argued to reinforce inequalities in some cases [22]. Nevertheless, understanding perceptions of different stakeholder groups towards FFLs may help clarify the culturally constructed role of gender in forestry, which can certainly help create opportunities for economic empowerment and development.

The invisibility of FFLs can be seen in Sharik et al. [5], who reported that women and minority student enrollment are lowest in the forestry discipline across the nation among all the disciplines related to natural resources. Similarly, Bal and Sharik [23] found that there exists a significant underrepresentation of women on forestry-major webpages, and even if they are covered, they are portrayed in a passive image, such as posing on campus rather than in an active image. Huff et al. [6] focused on the extension needs of FFLs. They reported that dedicated extension efforts and peer-to-peer learning opportunities could help FFLs in learning about the sustainable management of forest resources.

Other studies in the existing literature refer to FFLs, but only as a part of a broader investigation (Table 1). Bliss et al. [24] reported that a significantly lower number of FFLs approved the use of prescribed burning and herbicides. Sullivan et al. [25] found that female respondents were less interested in undertaking active forest management and harvesting activities than male respondents. Jarrett et al. [26] reported that FFLs were more likely to obtain forestry-related information from friends and family than male landowners, preferred informational pamphlets, and preferred state interventions and technical assistance. Sun et al. [27] found that application frequencies for a reforestation cost-share program were lower for FFLs, while Schelhas et al. [4] reported that FFLs were more likely to participate in cost-sharing programs than males. Miller et al. [28] reported that FFLs are less likely to participate in the forest carbon markets. Still, Thompson and Hansen [29] did not find any statistical difference between males landowners and FFLs for their positive or negative attitudes toward carbon sequestration and trading. Finally, Poudyal et al. [30] found that FFLs are less likely to convert their forestlands into other land uses and Hartter et al. [31] reported that women were more likely to express educational needs when it comes to forest management.

**Table 1:** Summary of the existing literature pertaining to female forest landowners.

Studies	Year	Area	Landowners (#)	Male	Female
Bliss et al. [24]	1992	Tennessee Valley Region	996	47%	53%
Sullivan et al. [25]	1999	Virginia	300	74%	26%
Jarrett et al. [26]	***	Southern United States*	585	72%	28%
Majumdar et al. [8]	2002–2006	United States	8373	79%	31%
Sun et al. [27]	2006	Mississippi	2229	74.9%	26.9%
Miller et al. [28]	2010	Lake States**	850	89%	11%
Schelhas et al. [4]	***	Alabama	235		
Thompson and Hansen [29]	2010	United States	429		
Poudyal et al. [30]	2007	Cumberland Plateau Tennessee	528	76%	24%
Hartter et al. [31]	2011	Oregon	1999	47.1%	52.9%
Butler et al. [7]	2011–2013	United States	1619	68.8%	31.2%

*Alabama, Florida, Georgia, Mississippi, South Carolina.

**Michigan, Minnesota, Wisconsin.

***Studies did not specify year/s that survey was distributed.

The current literature suggests that FFLs are inclined to manage their forestlands for conservation [32, 33], do not actively participate in federal and state incentive programs [27], seek forestry-related information from family and friends [4], and most importantly, are open to receive more information on various aspects of sustainable forest management including but not limited to reforestation, management, harvesting, taxes, and intergenerational land transfer [26]. These findings are in sync with other similar studies conducted on FFLs in Nordic countries [34–38], suggesting that FFLs share almost the same characteristics and related attitudes and behaviors across those developed countries which share a similar forestry landscape, i.e., a large portion of land is forested, a large wood product industry is active, and private forest landownership exists.

Generalizations about FFLs are based on studies that either treat gender as a variable in a regression equation with complete disregard about the process that generated the data related to the variable itself or have compared the differences between males and FFLs using extensive surveys over larger spatial scales. A need exists for descriptive studies that combine qualitative and quantitative approaches for developing a better understanding of FFLs. This will help in developing targeted extension programs and thereby promoting the active involvement of FFLs in the forestry sector. This will further help in encouraging forest stewardship among FFLs and ensuring the sustainability of forest resources in the United States. This is especially important as the percentage of forestland with FFLs is expected to increase in the coming decades with expected changes in demographics.

## 4) Study area

Considering the limitations imposed by resources and time, we focused on FFLs in Georgia, United States. In 2017, Georgia alone provided 3.9% of total roundwood harvested nationwide, the highest in the country. The total economic impact of Georgia’s forest industry was also generated $36.2 billion between 2017 and 2018 [39]. About 91% of the total forestland in Georgia is privately owned [40], out of which 57% belongs to family forest landowners [41]. FFLs own approximately 2.8 million acres [2] which equates to about 12% of the total forestland in Georgia [40]. Of these female-owned forested acres, about 47% (1.1 million acres [42]) are owned by a sole owner, while the remaining acres are under the shared ownership (two or more owners). The total forestland under female ownership will increase in Georgia in the foreseeable future, largely following the demographic trends at the regional and national levels.

## 5) SWOT-AHP analysis framework

The SWOT (strengths, weaknesses, opportunities, and threats) analysis is an established technique for understanding stakeholder perceptions by analyzing the internal and external environments that influence the perceptions of stakeholder groups [43, 44]. Internal factors are those that directly influence the study area and are comprised of strengths and weaknesses. External factors consist of opportunities and threats and have an indirect influence on the study area [45]. Factors that influence the perceptions of stakeholder groups are identified and categorized into a respective SWOT category based on stakeholder consultations.

The SWOT technique is commonly used to develop appropriate strategies that balance the identified internal and external factors [43, 46]. However, it cannot quantitatively analyze the factors [43, 46]. Additionally, it does not allow researchers to compare the factors to understand their priority rankings [46–48]. The SWOT analysis also does not allow the researchers to determine which factors are most important and how they should correlate in strategy development [49, 50].

The analytic hierarchy process (AHP) was introduced to the SWOT analysis technique for ensuring a quantitative prioritization of factors. Developed by Saaty [51], the AHP is a quantitative method used to analyze complex decision problems with multiple criteria [52]. The SWOT analysis acts as an ideal framework for AHP as it allows researchers to analytically examine the identified SWOT factors and compare them [53]. The SWOT-AHP technique allows for more precise strategy development as it grants researchers an opportunity to propose strategies that highlight the highest-ranking positive (strengths/opportunities) while addressing the highest-ranking negative (weaknesses/threat) factors [43].

A common three-step approach outlined by Kurttila et al. [43] is used by researchers.

- Factors are identified by researchers for each SWOT category [54, 55]. These factors can be identified through several means, including expert consultations, interviews with key stakeholders, and careful literature review.
- The AHP method is applied to determine the SWOT factor priority rankings [48]. Researchers do this by developing a survey or questionnaire to distribute to representatives from each stakeholder group included in the analysis [54]. The survey asks the stakeholder representatives to compare the identified factors to one another, within their respective SWOT category, through pairwise comparisons. These comparisons are then evaluated with the eigenvalue method [43, 47]. Each factor is given a priority ranking, also referred to as local priority rankings.
- The highest-ranking factor in each SWOT category, determined through the survey, is compared by stakeholder representatives through another survey that utilized pairwise comparisons [43]. Again, the eigenvalue method is used for the evaluation, and rankings are used to determine the overall global priority scores. These global priority scores, along with the local priority rankings, are used for strategy development.

The information from the pairwise comparisons can be represented using a reciprocal matrix where the appointed relative weights enter the matrix as α_ij,_ and the reciprocal (1/α_ij_) is assigned to the opposite side of the diagonal represented below.


$$A=(\alpha_{ij})[\begin{array}{cccc} w_{1}/w_{1} & w_{1}/w_{2} & \ldots & w_{1}/n\\ w_{2}/w_{1} & w_{2}/w_{2} & \ldots & w_{2}/n\\ \cdot & \cdot & \ldots & \cdot \\ \cdot & \cdot & \ldots & \cdot \\ \cdot & \cdot & \ldots & \cdot \\ w_{n}/w_{1} & w_{n}/w_{2} & \ldots & w_{n}/w_{n} \end{array}]$$

The rows of the matrix represent the ratios of weights for each factor in respect to all others. So, in the matrix where ^*i = j*^, then *α_ij_* = 1. We then multiply the matrix by the reverse of the vector weights, w, to develop a new vector *n***w**,

Aw=nw

where ***w*** *= (w*_*1*_,*w*_*2*_,*…*,*w*_*n*_*)*^*T*^ and *n* is the number of rows or number of columns. This can be written as

(A‐nI)w=0

where *n* is also the largest eigenvalue, λ_max_, or trace of matrix **A** and **I** is the identity matrix of size *n*—ensuring that λ_max_ = *n* is a necessary condition to confirm consistency. Inconsistency could arise if there are varying responses in the pairwise comparisons, which leads to λ_max_ deviating from *n*. The following formula should be used to test for the consistency of the matrix.

$$CI=(\mu_{max}‐n)/(n‐1)$$

CR=CI/RI

where **CI** is the consistency index, **RI** is the random index- created from a random matrix of order *n*, and **CR** is the consistency ratio. Saaty [51] identifies that the CR should be less than 10% to confirm consistency. The three things that improve CI are homogeneity of factors in each SWOT group, a smaller number of factors in each group, and a better understanding of the decision problem. For undertaking SWOT-AHP, large sample size is not needed; therefore, it helps in providing intrinsic motivations of stakeholder groups at a reasonable research expenditure.

## 6) Methods

### 6.1 Identify SWOT factors

We conducted a comprehensive literature review for identifying relevant factors under each SWOT category [4, 7, 8, 32, 36]. Then, we interviewed 14 Georgia-based professionals across four forestry stakeholder groups, i.e., FFLs (three interviews), Government Representatives (four interviews), Private Foresters (four interviews), and Non-profit Representatives (three interviews). The authors obtained approval (# PROJECT00002589) from the University of Georgia Institutional Review Board before undertaking the research. Out of 14 professionals, 8 were females. In these interviews, professionals were asked to review the identified factors based on the literature review. The interview also provided the stakeholders an opportunity to suggest other suitable factors that were not previously identified by researchers. Following these interviews, we finalized the factors under each SWOT category (Table 2).

**Table 2:** SWOT factors and their definitions used in the first survey.

Strengths	**Enhanced Environmental Services**: Increased participation of women forest landowners in forest management can improve water quality, increase carbon storage, enhance recreational opportunities, and better wildlife habitats.
	**Income Opportunities**: Increased participation of women forest landowners in forest management will provide additional income opportunities (e.g., hunting leases, thinning, harvesting, pine straw raking, etc.).
	**Participation in Existing Networks**: Bringing women forest landowners into forestry-related networks will add diverse perspectives and create women leaders at local and regional levels.
	**Connection to Land**: Active forest management will increase emotional and physical attachment to the land, thus motivating women landowners to retain ownership for themselves and for future generations.
Weaknesses	**Limited Knowledge of Forest Management**: Women forest landowners often possess limited knowledge about forestry and forest management, especially when buying or inheriting land.
	**Lack of Women-Centric Outreach Programs**: A lack of women-oriented forestry conferences/workshops for continued education in forest management and forestry practices places women owners at a disadvantage.
	**Limited Women Forest Professionals**: A lack of women forest professionals may limit a typical women forest landowner’s active participation in discussion of land management with a same-gendered counterpart.
	**Absence of Initial Contact**: When purchasing or inheriting land for forest management, women forest landowners may lack resources or access to professional consultations.
Opportunities	**Peer-to-Peer Educational Opportunities**: Participation in forest landowner-oriented educational conferences for women may facilitate experienced women forest landowners and women forestry professionals to educate, advise, and train other women forest landowners.
	**Community Development**: Forestry can facilitate community formation for discussing forests and related land management issues among women forest landowners, forestry professionals, and the broader forestry community.
	**Access to New Markets**: Active forest management is critical to enrolling forests in new markets for environmental services (e.g., carbon markets, water quality markets, stream mitigation banking, wetland mitigation banking, recreational opportunities, etc.).
	**Enhanced Job Opportunities for Women**: For various reasons, including assisting women forest landowners, women foresters are essential to a vibrant forestry industry.
Threats	**Investment Risks**: A fluctuating demand for forestry products and the potential of environmental risks such as hurricanes, wildfires, and pests can affect forest profitability.
	**Lack of Representation:** Women forest landowners are underrepresented in relevant government policy decisions.
	**Absenteeism**: When women inherit forest land, many may be absentee landowners which can lead to detachment from the land and a decision to sell the property.
	**Limited Interest from Future Generations**: If future generations’ interest in forest management is limited or nonexistent, parcellation of forestland may become more prevalent and retention of ownership threatened.

### 6.2 Data collection and analysis for survey one

A survey was developed based on the finalized factors, which utilized pairwise comparisons to compare and rank factors within each SWOT category. A workshop was conducted in Athens, Georgia, on October 1, 2020. The survey was distributed to the FFLs in attendance. Those FFLs who did not own land in Georgia were not included in the survey. In total, 11 FFLs filled out the survey at the workshop, and an additional response was received from an email survey totaling 12 FFL responses. Shortly following the workshop, a list of females from other stakeholder groups working in Georgia was compiled. In total, 15 private foresters, 17 government representatives, and 12 non-profit representatives were contacted through email to participate in the survey. Survey responses varied from each stakeholder group, with an average response rate of 60% (Table 3). Many non-profits were represented, including the Longleaf Alliance, Georgia Land Trust, Georgia Heirs Property, Southern Regional Extension Forestry, and the Georgia Nature Conservancy. Government representatives were from the Georgia Department of Natural Resources (DNR), the Georgia Forestry Commission (GFC), and the United States Department of Agriculture (USDA). A collection of private companies was represented in the private stakeholder group, including International Paper and Weyerhaeuser.

**Table 3:** Response rate for the first SWOT survey by selected stakeholder groups.

	Landowners	Non-profit	Government	Private Foresters	Total
**Surveys Sent (#)**	12	11	17	15	55
**Surveys Received (#)**	12	7	8	6	33
**Response Rate**	100%	63.6%	47.1%	40%	60%

### 6.3 Data collection and analysis for survey two

For each stakeholder group, researchers analyzed the geometric means from the survey responses to determine the highest priority factors for each SWOT category. These scores were used to create four more surveys, one for each stakeholder group, that used pairwise comparisons to determine which factor and SWOT category were most important to each stakeholder group. The surveys were individually emailed to all first survey respondents. Additional landowner surveys were distributed and collected at the second workshop in Albany, Georgia, on February 18, 2021. Stakeholder responses varied but averaged 66.6% (Table 4). Necessary precautions were taken to ensure that each stakeholder was given the correct survey. This was especially important as the highest priority values varied between stakeholder groups. Responses from individuals for each stakeholder group were combined to determine the geometric means of all pairwise comparisons. The relative priority for each category in each stakeholder group was derived from the geometric means of SWOT categories using the standard AHP technique. All CRs were below 10% to ensure consistency, as outlined by Saaty [51].

**Table 4:** Response rate for the second round of SWOT surveys.

	Landowners	Non-profit	Government	Private Foresters	Total
**Surveys Sent (#)**	21	7	8	6	42
**Surveys Received (#)**	10	5	7	6	28
**Response Rate**	47.6%	71.4%	87.5%	100%	66.6%

## 7) Results and discussions

The results section is divided into three separate sub-sections. The first section focuses on the local factor priorities from the first survey. The second section discusses the global factor priorities obtained from the second survey distributed to each stakeholder group. The third section elaborates on the overall priority scores for each factor.

### 7.1 Local priority scores

**Fig 2:**
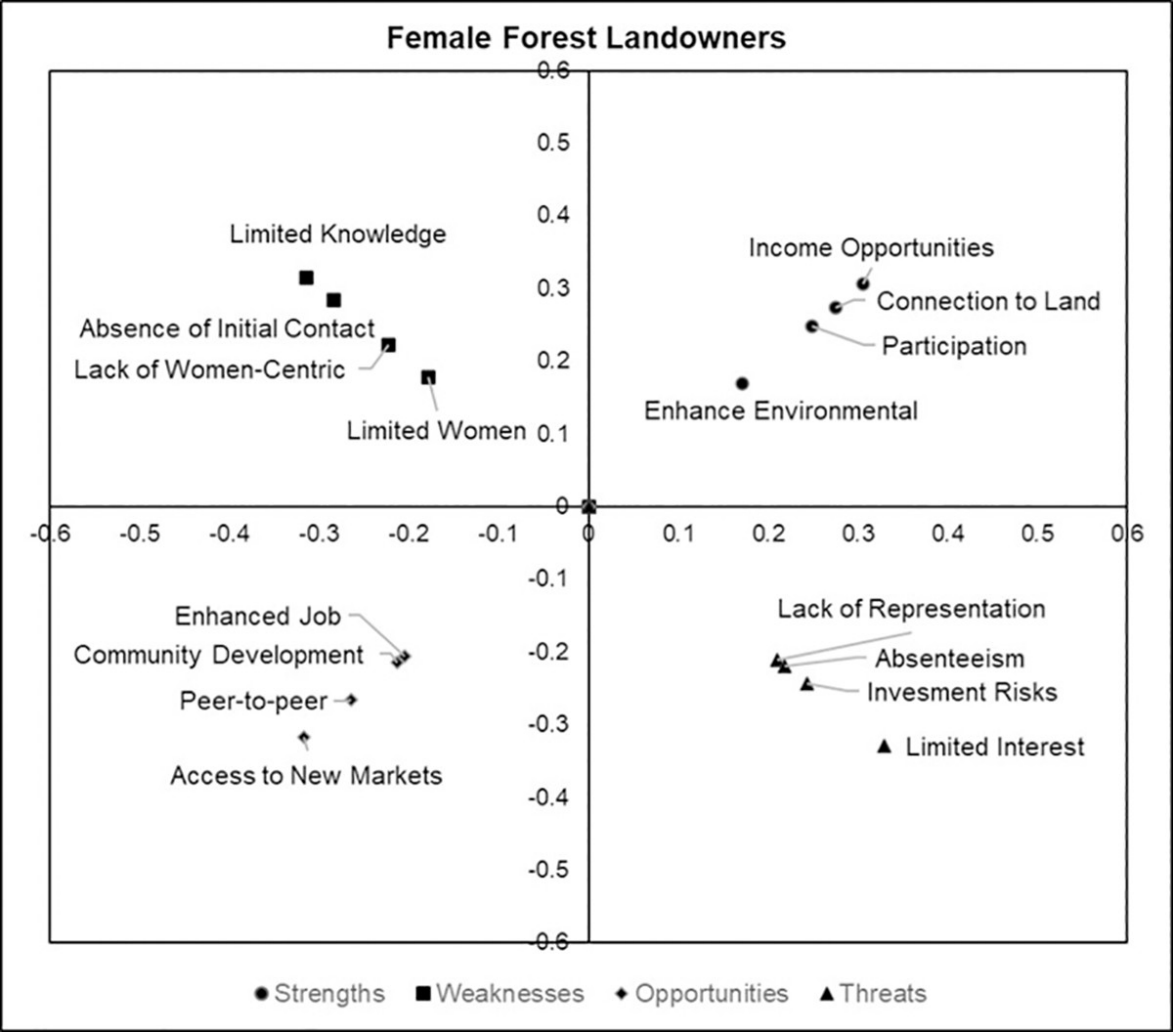
Perception map of factor priorities assigned by female forest landowners.

**Fig 3:**
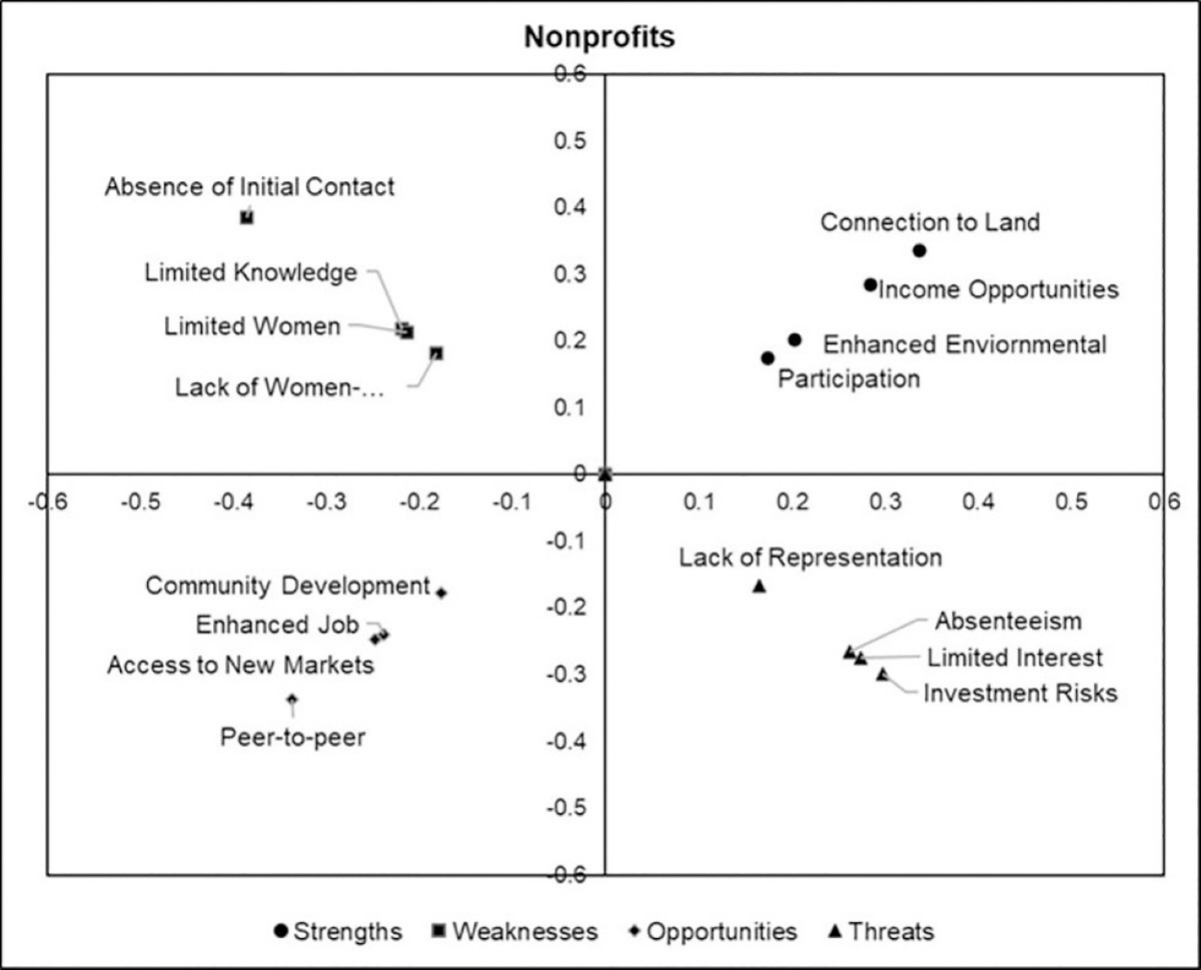
Perception map of factor priorities assigned by non-profit representatives.

**Fig 4:**
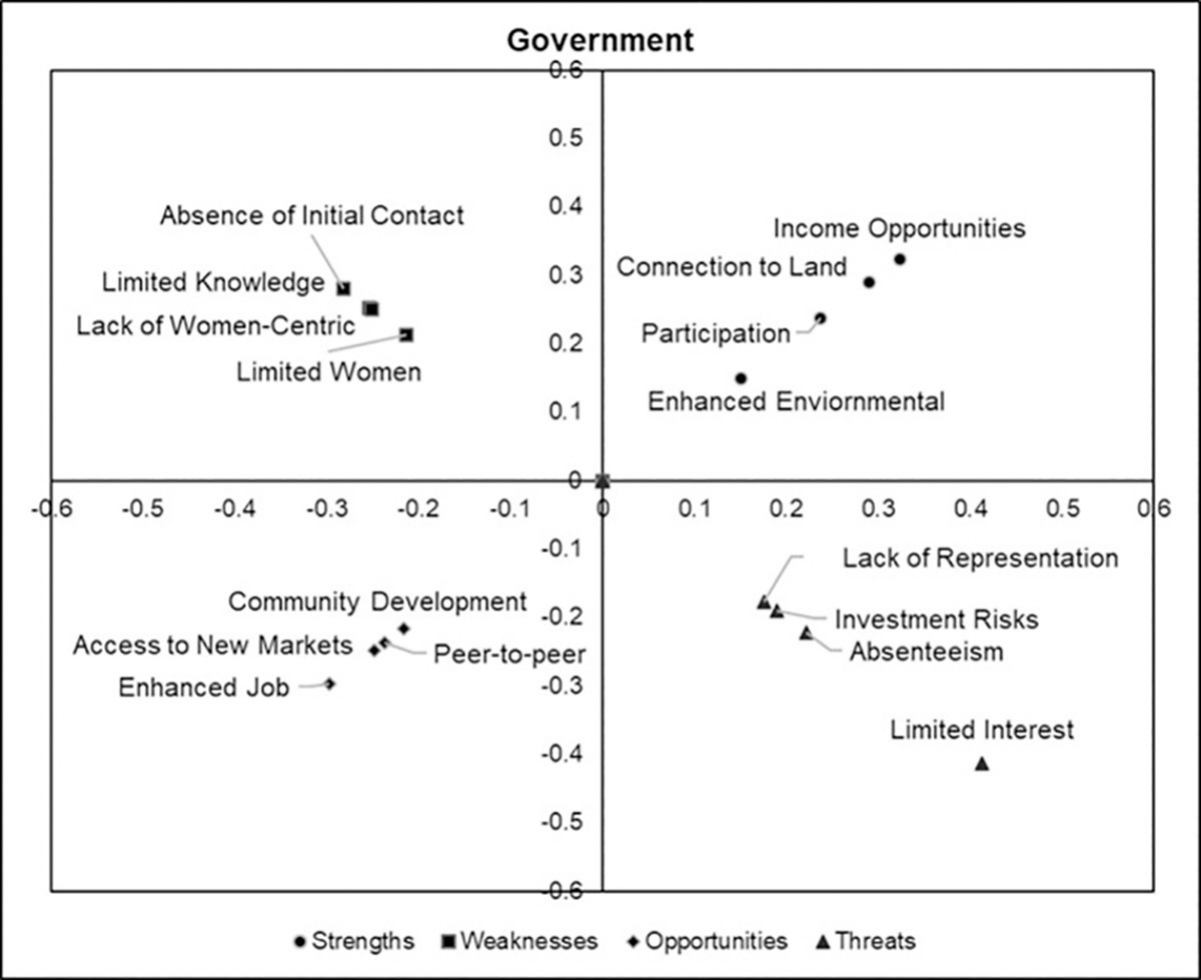
Perception map of factor priorities assigned by government representatives.

**Fig 5:**
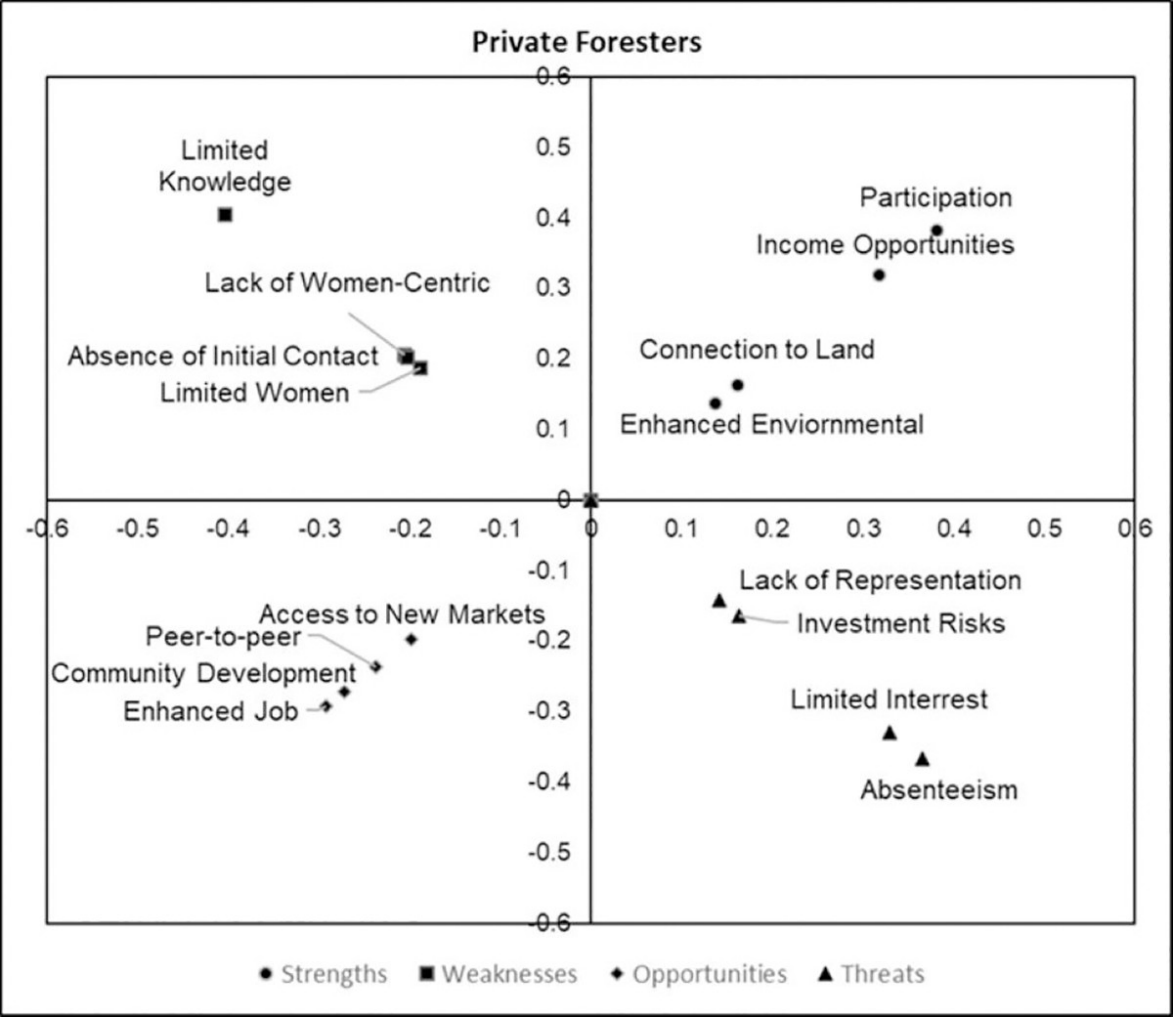
Perception map of factor priorities assigned by private foresters.

Figs 2–5 are perceptions maps derived from the geometric mean obtained after analyzing the data collected from the first survey for each stakeholder group. It should be noted that factors furthest from the origin were assigned the highest priority while the factors nearest to the origin were given the least priority.

For the FFL stakeholder group, the strengths category was led by *income opportunities* (30.6%), followed by *connection to the land* (27.4%), *participation in existing networks* (24.9%), and *enhanced environmental services* (17%). The two factors that received the highest priority score for weaknesses were *limited knowledge of forest management* (31.5%) and *absence of initial contact* (28.4%). These two factors encompass approximately 60% of FFLs’ perception of forestland management weaknesses. *Access to new markets* (31.7%) dominated opportunities, while *limited interest from future generations* (32.9%) most defined the threats category (Fig 2).

For the non-profit stakeholder group, the members of this stakeholder group assigned the highest priority scores to *connection to land* (33.7%) and *income opportunities* (28.5%) among all the factors present under the strengths category. They dominantly ranked *absence of initial contact* (38.6%) over *limited knowledge of forest management* (21.8%), *limited women forest professionals* (21.3%), and *lack of women-centric outreach programs* (18.2%). The opportunities category was most defined by *peer-to-peer educational opportunities* (33.7%) and *investment risks* (29.8%) under the threats category (Fig 3).

Government representatives prioritized *income opportunities* (32.3%) and *connection to land* (28.9%) over the other factors under the strengths category. Like the non-profit stakeholder group, the government representatives ranked *absence of initial contact* (28.2%) and *limited knowledge of forest management* (25.3%) the highest. However, it should be noted that the *lack of women-centric outreach programs* (25.1%) ranked almost equally to *limited knowledge of forest management* (25.3%). The government representatives showed slight preference over factors in the opportunities category, shown by the low variability in the ranking of the factors. The highest priority factor was *enhanced job opportunities for women* (29.8%), followed by *access to new markets* (24.9%), *peer-to-peer educational opportunities* (23.8%), and *community development* (21.6%). Like FFLs, the *limited interest of future generations* (41.3%) was dominantly prioritized by the government representatives (Fig 4).

Fifteen private foresters were contacted through email to complete the first survey. Their overall rankings are shown in Fig 5. Of these 15 individuals, six responded, providing a 40% response rate (Table 3). Unlike the other stakeholder groups, the private foresters prioritized *participation in existing networks* (38.2%) over the other factors in the strength category. However, *income opportunities* (31.8%) ranked second, similar to the other stakeholder groups. Like the FFLs, the private foresters prioritized *limited knowledge of forest management* (40.4%) over the other factors in the weaknesses category. Like the government stakeholder group, the private foresters prioritized *enhanced job opportunities for women* (29.2%) for opportunities. *Absenteeism* (36.6%) was given the highest rank in the threats, followed by *limited interest from future generations* (32.9%), *investment risks* (16.1%), and *lack of representation* (14.1%).

### 7.2 Global priority scores

**Fig 6:**
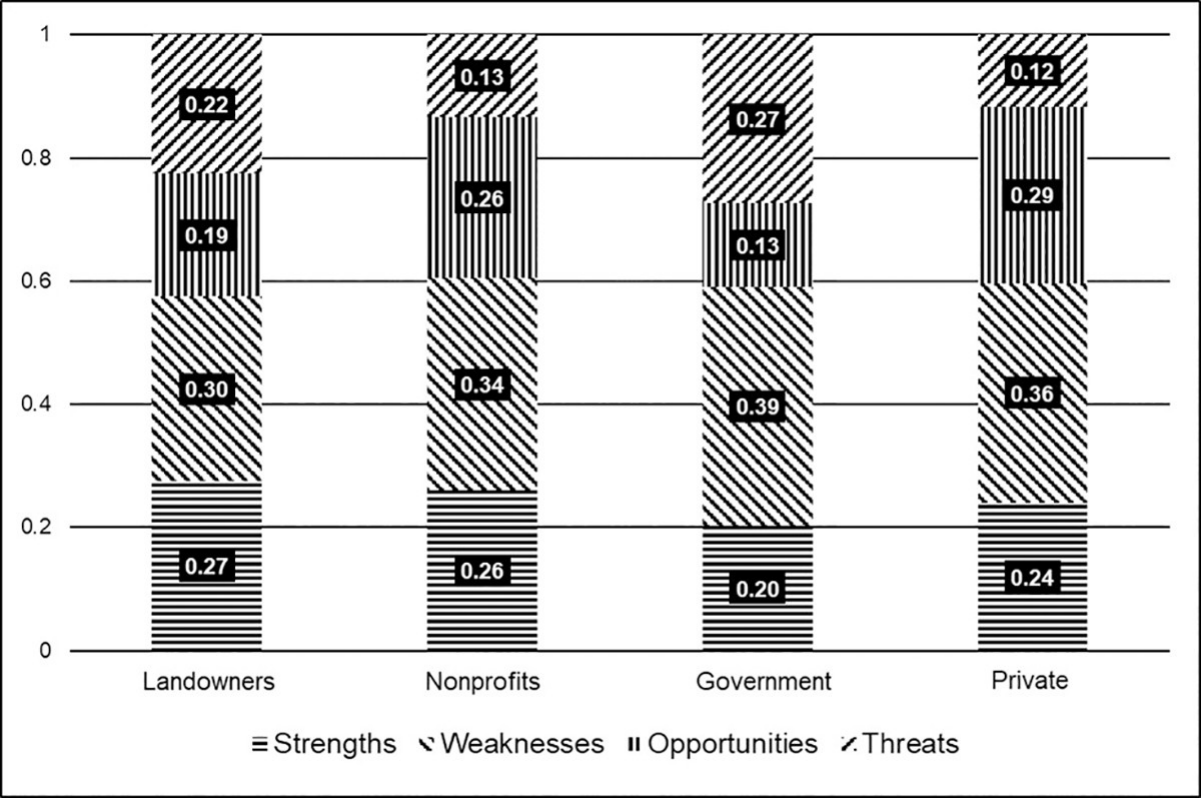
SWOT category factor priorities for each stakeholder group.

Fig 6 displays the breakdown of how each stakeholder group prioritized the SWOT categories. These priority scores were obtained from the data collected from the second survey. Individuals from each group compared the highest-scoring factor from each SWOT category through pairwise comparisons. This gave us an idea of what SWOT category was most important to each group. This figure allows us to identify the overall perception, positive or negative, for each stakeholder group. For SWOT analyses, strengths and opportunities can be combined as positive factor priorities, while weaknesses and threats can be combined as negative factor priorities.

Negative perceptions were held by FFLs (-52%) and government representatives (-66%), while private foresters (+53%) and non-profits (+52%) had slightly positive perceptions. The government stakeholder group had the most negative perception. They ranked weaknesses (39%) and threats (27%) highest over strengths (20%) and opportunities (13%). FFLs prioritized weaknesses (30%) followed by strengths (27%), highlighting their more neutral but still slightly negative perception. Though private foresters and non-profit representatives prioritized weaknesses (36% and 34%, respectively) first, they also highly ranked opportunities (29% and 26%, respectively) and strengths (24% and 26%, respectively); thus, leading to their overall more positive perception.

The high priority score given to weaknesses by all stakeholder groups demonstrates a hesitation present regarding forestland management for FFLs. This can be attributed to the lack of extension efforts available to current and future FFLs. This is stressed by our results as FFLs and private foresters prioritized *limited knowledge of forest management* over other factors in the weakness category. This underscores the importance of educating FFLs in forestland management through extension efforts by government agencies, non-profit organizations, and private industry. Providing educational opportunities catered to FFLs may provide women more comfortable environments to learn about forestland management options [6]. Additionally, the non-profit and government stakeholder groups prioritized a *lack of initial contact* for the weaknesses. Not only could these educational opportunities better inform current and future FFLs about forestland management options, but they could also strengthen the connection of FFLs with each other and individuals in the forestry industry.

Non-profit representatives felt that *peer-to-peer educational opportunities* was the most important opportunity factor, further emphasizing the need for enhanced educational outreach. Providing educational opportunities catered towards FFLs will not only expand their knowledge of forestland management, but it will also create an environment to foster connections with other FFLs. These connections with other landowners could help them make integral management decisions for their forestland, which is especially important to ensure that the Southern United States maintains its position as the wood basket of the world. This supports conclusions drawn by Schubert and Mayer [56], who found that about half of family forest landowners were influenced by members of their forest communities in the Western Upper Peninsula of Michigan. Additionally, other studies [57, 58] found that while professional consultations are important in forest landowner management decisions, landowners are also influenced by peer landowners. Though overall feelings towards forestland management for FFLs are negative due to flaws in the current outreach system, there are groups (non-profit representatives and private foresters) who believe in the opportunities provided by increasing FFLs and demonstrates room to grow in forestry.

On the other hand, the government representatives and FFLs held negative perceptions of forestland management for FFLs. The government representatives ranked threats as the second most important category next to weaknesses, while the FFLs ranked strengths second and threats third. Both groups prioritized the *limited interest of future generations* as the most influential threat factor. During our interviews with representatives of stakeholder groups, the importance of engaging future generations in forestry was brought up often. One government representative felt that it was not so much of a lack of interest in forestry as there is a disconnect between the interest of FFLs, especially younger women landowners, and conventional forestry practices. This further emphasizes the importance of educational outreach opportunities and how essential they are for women inheriting or purchasing their forestland now and well into the future.

### 7.3 Overall priority scores

**Fig 7:**
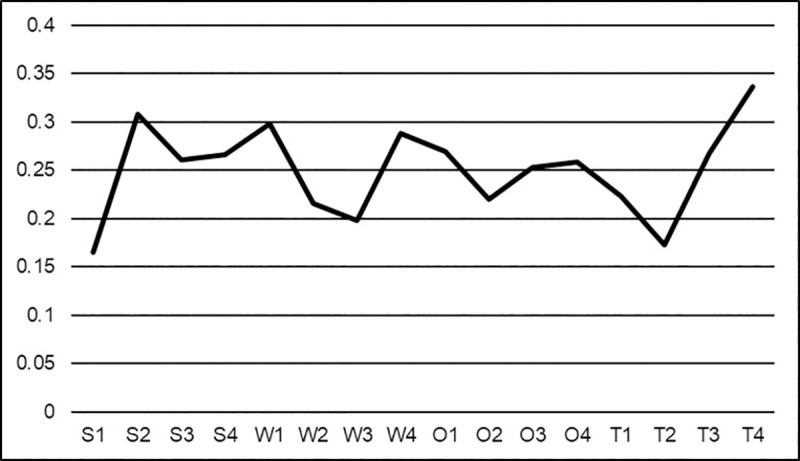
The overall average priority scores of each SWOT factor from the first survey. S1: enhanced environmental services, S2: income opportunities, S3: participation in existing networks, S4: connection to land, W1: limited knowledge of forest management, W2: lack of women-centric outreach programs, W3: limited women forest professionals, W4: absence of initial contact, O1: peer-to-peer educational opportunities, O2: community development, O3: access to new markets, O4: enhanced job opportunities for women, T1: investment risks, T2: lack of representations, T3: absenteeism, T4: limited interest from future generations.

Priority scores assigned by each stakeholder group in addition to the average for each factor was taken to determine the overall priority scores of each individual factor (Fig 7). Overall, *income opportunities* (30.8%) was given the highest priority for strengths. This is followed by *connection to land* (26.6%) and *participation in existing networks* (26.05%). The overall highest ranked weakness was *limited knowledge of forest management* (29.8%), closely followed by an *absence of initial contact* (28.9%). Overall priority scores for opportunities were very close. The most prioritized factor was *peer-to-peer educational opportunities* at 26.9%, followed by *enhanced job opportunities for women* (25.8%) and *access to new markets* (25.3%). *Limited interest from future generations* (33.6%) was ranked as the overall most important factor for threats and was followed by *absenteeism* (26.7%) and *investment risks* (22.3%).

The high priority given to *limited interest from future generations* indicates that all stakeholder groups view the decreased interest in forestry by younger generations as the most significant threat to forestland management. The lack of engagement from future generations is highlighted by Sharik et al. [5], who found that although undergraduate enrollment in natural resource programs is steadily increasing, there has been a decline in enrollment in forestry. One of the biggest concerns for landowners throughout the United States is keeping land intact for heirs [41, 59]. This may explain why FFLs prioritized this factor over all other threat factors. The factor definition (Table 2) implied increased parcellation of land if future generations continue to express a lack of interest in forestry. This is an especially prominent issue for landowners in the Southern United States as these landowners are more likely to have inherited their land compared to other regions in the United States [60].

The second-highest-ranking factor *income opportunities* suggest that the stakeholder groups are most excited by the economic opportunities provided by active forestland management for FFLs. However, the stakeholder groups seem most intrigued by more traditional forest products as *access to new markets* only ranked tenth overall. This seems to support conclusions drawn that forest landowners in the Southern United States are more likely to manage their land for consumptive purposes [60]. Additionally, the factor *investment risks* is lowly ranked, which further proves that FFLs in Georgia are more excited about managing their forestland for profit despite the possible investment risk. This is also supported by *enhanced environmental services* being ranked lowest overall by the stakeholder groups. This finding does not support the conclusion other studies [32, 33] have found where women tend to manage their land for conservation. This difference could be attributed to the fact that the Southern United States is the largest supplier of roundwood in the United States, where a significant percentage of family forest landowners harvest their forestlands regularly for income generation [1].

The stakeholder groups also felt that the biggest challenge facing FFLs is their limited knowledge of forest management and their lack of resources or access to professional consultants. This is indicated by the high overall priority scores of *limited knowledge of forest management* and the *absence of initial contact*. This supports the findings of Butler et al. [59] and Schelhas et al. [4], which state that most forest landowners want some type of information for their forestland. Our data also suggests that women prefer to receive this knowledge and these contacts through their peers. *Peer-to-peer educational opportunities* was the highest-ranking information transfer factor by all stakeholder groups. Butler et al. [59] determined that the most popular advice topics include timber management which additionally supports our findings on the economic incentive that forestland provides to landowners.

Finally, all stakeholder groups agreed that *lack of representation* was the least important threat. All four stakeholder groups ranked this factor behind all others, and all groups rated the priority 21% or less. Landowners seemed to be the most worried about the lack of representation of female forest landowners in government policy decisions as they prioritized it at 20.9% of all threats. In comparison, all others prioritized it at 17.6% or less. Despite this being a low ranking factor, the effect of low numbers of female forest landowners in positions to influence government policy may have detrimental effects not only on their land but also on other forestlands owned by families.

**Fig 8:**
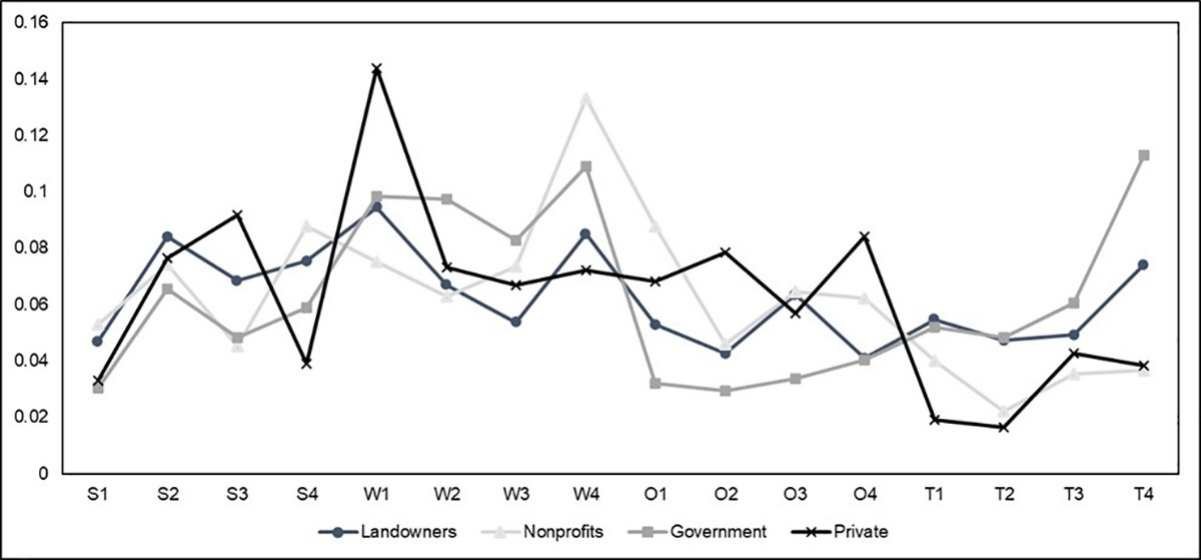
Overall perceptions of SWOT factors by stakeholder group. S1: enhanced environmental services, S2: income opportunities, S3: participation in existing networks, S4: connection to land, W1: limited knowledge of forest management, W2: lack of women-centric outreach programs, W3: limited women forest professionals, W4: absence of initial contact, O1: peer-to-peer educational opportunities, O2: community development, O3: access to new markets, O4: enhanced job opportunities for women, T1: investment risks, T2: lack of representations, T3: absenteeism, T4: limited interest from future generations.

The overall category priority scores (global scores) obtained from the second survey were multiplied by the individual scores (local scores) obtained from the first survey. Fig 8 displays how individual stakeholder groups prioritized the SWOT factors. We can see similar patterns between the FFLs, non-profit representatives, and government representatives. These three groups prioritize *income opportunities* (S2) and *connection to land* (S4) over the other factors in the strengths category, whereas the private foresters prioritized *participation in existing networks* (S3). For weaknesses, we see the FFLs, non-profit, and government stakeholder groups significantly prioritizing *absence of initial contact* (W4) while the private foresters significantly prioritized *limited knowledge of forest management* (W1) and ranked *absence of initial contact (W4)* third in priority behind *lack of women-centric outreach programs* (W2). We see the most discrepancy between stakeholders in the opportunities category. Private foresters ranked *enhanced job opportunities for women* (O4) the highest, while non-profits prioritized *peer-to-peer educational opportunities* (O1). Government representatives had little difference in their rankings of the factors but slightly prioritized *enhanced job opportunities for women* (O4) like the private foresters. FFLs ranked *access to new markets* (O3) highest, unlike any other stakeholder group. While all stakeholder groups ranked *limited interest from future generations* (T4) highly, government representatives and FFLs ranked it highest. Non-profits slightly out-prioritized T4 with *investment risks* (T1), and private foresters thought *absenteeism* (T3) was the biggest threat. This further emphasized the difference between priority rankings between private foresters and the other stakeholder groups as we observed a slightly positive perception for the private forester group compared to the other stakeholder groups who had more negative perceptions.

## 8) Conclusion

This paper utilized SWOT-AHP to analyze the perceptions of four stakeholder groups about FFLs in Georgia, United States. This state was chosen due to the high prevalence of FFLs as well as the high production of roundwood and finished wood-based products. Despite the study focusing on one state, the results can be utilized by other southern states due to similar social, cultural, and policy conditions.

Our results highlight differences between stakeholder perceptions of forestland management for FFLs. While perceptions of all stakeholder groups perceptions were dominated by weaknesses, the government stakeholder group held the most negative perception, followed by FFLs who had only a slightly negative perception of forestland management. On the other hand, the non-profit and private forester stakeholder groups saw the potential opportunity of forestland management for FFLs; thus, explaining their more positive perception.

Overall, our analysis demonstrates a vital need for educational opportunities for FFLs. Literature suggests that professional advice and peer-to-peer learning are viable options for knowledge transfer for new and future forest landowners. Our findings suggest that FFLs in Georgia see their limited knowledge of forestland management as their biggest challenge when inheriting or purchasing land despite their excitement for the income opportunities available with forestland. We also see that the non-profit and FFLs stakeholder groups see peer-to-peer educational opportunities as one of the most important opportunities for FFLs. Results from our overall priority scores suggest that stakeholders are most concerned about the limited interest from future generations when it comes to forestland management. This is not surprising as many individuals in our interviews discussed this as a threat to the future of forestry. Additionally, the stakeholders felt that the greatest strength for FFLs in forestland management was economic gain and return from traditional forest products such as timber. This finding differs from other existing studies as they found women manage their forestland for more environmental benefits and tend to be more environmentally conscious. The stakeholders felt that the biggest weakness for FFLs was their lack of knowledge about forestland management and their limited access to professional consultants, which further emphasizes our need to generate educational opportunities and outreach programs which cater to women.

The results from this study represent aggregated opinions of respondents that belong to the four chosen stakeholder groups. Despite the consistent CRs, results were dependent on individuals’ responses which could lead to results that are not representative of all stakeholder opinions. This emphasizes a need for research that incorporates more stakeholder groups (e.g., industry). This will allow a clearer understanding of more comprehensive stakeholder perceptions for more intentional educational efforts by relevant government, non-profit, and private groups.

We hope this research will help fill in existing gaps in literature around FFLs in the United States and will increase awareness of the challenges and opportunities present for FFLs in forestland management. The research contributes to existing literature that investigates FFLs and forestland management. This information and methodology can be utilized in ongoing research of stakeholder perceptions of forestland management. Though our paper investigated the relevant stakeholder perceptions of forestland management for FFLs, we did not do any research which was relevant to gender differences in these perceptions. Further research could explore gendered stakeholder perceptions FFLs forestland management. Additionally, this research focused on just Georgia, United States. This methodology and research can be utilized to research other regions in the United States and other countries throughout the world to provide a more comprehensive understanding of stakeholder perceptions of FFLs about forestland management.

## Supporting information

S1 File First survey distributed to FFLs, private foresters, government and non-profit representatives. (PDF)

S2 File Second survey distributed to government representatives. (PDF)

S3 File Second survey distributed to FFLs. (PDF)

S4 File Second survey distributed to non-profit (or NGO) representatives. (PDF)

S5 File Second survey distributed to individuals in private industry. (PDF)
