# Supplementary material for: Perspectives of four stakeholder groups about the participation of female forest landowners in forest management in Georgia, United States
Source: PLoS One. 2021 Aug 24;16(8):e0256654. doi: 10.1371/journal.pone.0256654 (PMC8384192; doi:10.1371/journal.pone.0256654)
Supplement: S1 File — (PDF) [file pone.0256654.s001.pdf]

Identified Factors and Their Definitions

As you complete the survey, you may find it helpful to refer to this page to clarify terms used in the survey portion.

Strengths

Enhanced Environmental Services: Increased participation of women forest landowners in forest management can improve water quality, increase carbon storage, enhance recreational opportunities, and better wildlife habitats.

Income Opportunities: Increased participation of women forest landowners in forest management will provide additional income opportunities (e.g., hunting leases, thinning, harvesting, pine straw raking, etc.).

Participation in Existing Networks: Bringing women forest landowners into forestry-related networks will add diverse perspectives and create women leaders at local and regional levels.

Connection to Land: Active forest management will increase emotional and physical attachment to the land, thus motivating women landowners to retain ownership for themselves and for future generations.

Weaknesses

Limited Knowledge of Forest Management: Women forest landowners often possess limited knowledge about forestry and forest management, especially when buying or inheriting land.

Lack of Women-Centric Outreach Programs: A lack of women-oriented forestry conferences/workshops for continued education in forest management and forestry practices places women owners at a disadvantage.

Limited Women Forest Professionals: A lack of women forest professionals may limit a typical women forest landowner's active participation in discussion of land management with a same-gendered counterpart.

Absence of Initial Contact: When purchasing or inheriting land for forest management, women forest landowners may lack resources or access to professional consultations.

Opportunities

Peer-to-Peer Educational Opportunities: Participation in forest landowner-oriented educational conferences for women may facilitate experienced women forest landowners and women forestry professionals to educate, advise, and train other women forest landowners.

Community Development: Forestry can facilitate community formation for discussing forests and related land management issues among women forest landowners, forestry professionals, and the broader forestry community.

Access to New Markets: Active forest management is critical to enrolling forests in new markets for environmental services (e.g. carbon markets, water quality markets, stream mitigation banking, wetland mitigation banking, recreational opportunities, etc.).

Enhanced Job Opportunities for Women: For various reasons, including assisting women forest landowners, women foresters are essential to a vibrant forestry industry.

Threats

Investment Risks: A fluctuating demand for forestry products and the potential of environmental risks such as hurricanes, wildfires, and pests can affect forest profitability.

Lack of Representation: Women forest landowners are underrepresented in relevant government policy decisions.

Absenteeism: When women inherit forest land, many may be absentee landowners which can lead to detachment from the land and a decision to sell the property.

Limited Interest from Future Generations: If future generations' interest in forest management is limited or nonexistent, parcelization of forestland may become more prevalent and retention of ownership threatened.

Paired Comparisons Between Identified Factors

Category: Strengths

Factor	Very Important	Important	Moderately Important	Equal	Moderately Important	Important	Very Important	Factor
Enhanced Environmental Services								Income Opportunities
Enhanced Environmental Services								Participation in Existing Networks
Enhanced Environmental Services								Connection to Land
Income Opportunities								Participation in Existing Networks
Income Opportunities								Connection to Land
Participation in Existing Networks								Connection to Land

Category: Weaknesses

Factor	Very Important	Important	Moderately Important	Equal	Moderately Important	Important	Very Important	Factor
Limited Knowledge of Forest Management								Lack of Women-Centric Outreach Programs
Limited Knowledge of Forest Management								Limited Women Forest Professionals
Limited Knowledge of Forest Management								Absence of Initial Contact
Lack of Women-Centric Outreach Programs								Limited Women Forest Professionals
Lack of Women-Centric Outreach Programs								Absence of Initial Contact
Limited Women Forest Professionals								Absence of Initial Contact

Category: Opportunities

Factor	Very Important	Important	Moderately Important	Equal	Moderately Important	Important	Very Important	Factor
Peer-to-Peer Educational Opportunities								Community Development
Peer-to-Peer Educational Opportunities								Access to New Markets
Peer-to-Peer Educational Opportunities								Enhanced Job Opportunities for Women
Community Development								Access to New Markets
Community Development								Enhanced Job Opportunities for Women
Access to New Markets								Enhanced Job Opportunities for Women

Category: Threats

Factor	Very Important	Important	Moderately Important	Equal	Moderately Important	Important	Very Important	Factor
Investment Risks								Lack of Representation
Investment Risks								Absenteeism
Investment Risks								Limited Interest from Future Generations
Lack of Representation								Absenteeism
Lack of Representation								Limited Interest from Future Generations
Absenteeism								Limited Interest from Future Generations

Please indicate your stakeholder group.

- ☐ Landowner
- ☐ Non-Profits
- ☐ Forester (Federal or State Agency)
- ☐ Forester (Private)
- ☐ Other (Please specify): ____________________

What does sustainable forestry mean to you? *(Please write down your response.)*

Thank you for participating in the survey!
