# Supplementary material for: Perspectives of four stakeholder groups about the participation of female forest landowners in forest management in Georgia, United States
Source: PLoS One. 2021 Aug 24;16(8):e0256654. doi: 10.1371/journal.pone.0256654 (PMC8384192; doi:10.1371/journal.pone.0256654)
Supplement: S2 File — (PDF) [file pone.0256654.s002.pdf]

Identified Factors and Their Definitions

As you complete the survey, you may find it helpful to refer to this page to clarify terms used in the survey portion.

Strength

Income Opportunities: Increased participation of women forest landowners in forest management will provide additional income opportunities (e.g., hunting leases, thinning, harvesting, pine straw raking, etc.).

Weakness

Absence of Initial Contact: When purchasing or inheriting land for forest management, women forest landowners may lack resources or access to professional consultations.

Opportunity

Enhanced Job Opportunities for Women: For various reasons, including assisting women forest landowners, women foresters are essential to a vibrant forestry industry.

Threat

Limited Interest from Future Generations: If future generations' interest in forest management is limited or nonexistent, parcelization of forestland may become more prevalent and retention of ownership threatened.

Paired Comparison Between Identified Factors

Government Pairwise Comparisons

Factor	Very Important	Important	Moderately Important	Equal	Moderately Important	Important	Very Important	Factor
Income Opportunities								Absence of Initial Contact
Income Opportunities								Enhanced Job Opportunities for Women
Income Opportunities								Limited Interest from Future Generations
Absence of Initial Contact								Enhanced Job Opportunities for Women
Absence of Initial Contact								Limited Interest from Future Generations
Enhanced Job Opportunities for Women								Limited Interest from Future Generations

Please indicate your stakeholder group.

- ☐ Landowner
- ☐ Non-Profits
- ☐ Forester (Federal or State Agency)
- ☐ Forester (Private)
- ☐ Other (Please specify): ____________________

Thank you for participating in the survey!
