# Supplementary material for: Perspectives of four stakeholder groups about the participation of female forest landowners in forest management in Georgia, United States
Source: PLoS One. 2021 Aug 24;16(8):e0256654. doi: 10.1371/journal.pone.0256654 (PMC8384192; doi:10.1371/journal.pone.0256654)
Supplement: S3 File — (PDF) [file pone.0256654.s003.pdf]

Identified Factors and Their Definitions

As you complete the survey, you may find it helpful to refer to this page to clarify terms used in the survey portion.

Strength

Income Opportunities: Increased participation of women forest landowners in forest management will provide additional income opportunities (e.g., hunting leases, thinning, harvesting, pine straw raking, etc.).

Weakness

Limited Knowledge of Forest Management: Women forest landowners often possess limited knowledge about forestry and forest management, especially when buying or inheriting land.

Opportunity

Access to New Markets: Active forest management is critical to enrolling forests in new markets for environmental services (e.g. carbon markets, water quality markets, stream mitigation banking, wetland mitigation banking, recreational opportunities, etc.).

Threat

Limited Interest from Future Generations: If future generations' interest in forest management is limited or nonexistent, parcelization of forestland may become more prevalent and retention of ownership threatened.

Paired Comparison Between Identified Factors

Landowner Pairwise Comparisons

Factor	Very Important	Important	Moderately Important	Equal	Moderately Important	Important	Very Important	Factor
Income Opportunities								Limited Knowledge of Forest Management
Income Opportunities								Access to New Markets
Income Opportunities								Limited Interest from Future Generations
Limited Knowledge of Forest Management								Access to New Markets
Limited Knowledge of Forest Management								Limited Interest from Future Generations
Access to New Markets								Limited Interest from Future Generations

Please indicate your stakeholder group.

- ☐ Landowner
- ☐ Non-Profits
- ☐ Forester (Federal or State Agency)
- ☐ Forester (Private)
- ☐ Other (Please specify): ____________________

Thank you for participating in the survey!
