# Supplementary material for: Perspectives of four stakeholder groups about the participation of female forest landowners in forest management in Georgia, United States
Source: PLoS One. 2021 Aug 24;16(8):e0256654. doi: 10.1371/journal.pone.0256654 (PMC8384192; doi:10.1371/journal.pone.0256654)
Supplement: S4 File — (PDF) [file pone.0256654.s004.pdf]

Identified Factors and Their Definitions

As you complete the survey, you may find it helpful to refer to this page to clarify terms used in the survey portion.

Strength

Connection to Land: Active forest management will increase emotional and physical attachment to the land, thus motivating women landowners to retain ownership for themselves and for future generations.

Weakness

Absence of Initial Contact: When purchasing or inheriting land for forest management, women forest landowners may lack resources or access to professional consultations.

Opportunity

Peer-to-Peer Educational Opportunities: Participation in forest landowner-oriented educational conferences for women may facilitate experienced women forest landowners and women forestry professionals to educate, advise, and train other women forest landowners.

Threat

Investment Risks: A fluctuating demand for forestry products and the potential of environmental risks such as hurricanes, wildfires, and pests can affect forest profitability.

Paired Comparison Between Identified Factors

Non-Governmental Organization (NGO) Pairwise Comparisons

Factor	Very Important	Important	Moderately Important	Equal	Moderately Important	Important	Very Important	Factor
Connection to Land								Absence of Initial Contact
Connection to Land								Peer-to-Peer Educational Opportunities
Connection to Land								Investment Risks
Absence of Initial Contact								Peer-to-Peer Educational Opportunities
Absence of Initial Contact								Investment Risks
Peer-to-Peer Educational Opportunities								Investment Risks

Please indicate your stakeholder group.

- ☐ Landowner
- ☐ Non-Profits
- ☐ Forester (Federal or State Agency)
- ☐ Forester (Private)
- ☐ Other (Please specify): ____________________

Thank you for participating in the survey!
