# Supplementary material for: Perspectives of four stakeholder groups about the participation of female forest landowners in forest management in Georgia, United States
Source: PLoS One. 2021 Aug 24;16(8):e0256654. doi: 10.1371/journal.pone.0256654 (PMC8384192; doi:10.1371/journal.pone.0256654)
Supplement: S5 File — (PDF) [file pone.0256654.s005.pdf]

Identified Factors and Their Definitions

As you complete the survey, you may find it helpful to refer to this page to clarify terms used in the survey portion.

Strength

Participation in Existing Networks: Bringing women forest landowners into forestry-related networks will add diverse perspectives and create women leaders at local and regional levels.

Weakness

Limited Knowledge of Forest Management: Women forest landowners often possess limited knowledge about forestry and forest management, especially when buying or inheriting land.

Opportunity

Enhanced Job Opportunities for Women: For various reasons, including assisting women forest landowners, women foresters are essential to a vibrant forestry industry.

Threat

Absenteeism: When women inherit forest land, many may be absentee landowners which can lead to detachment from the land and a decision to sell the property.

Paired Comparisons Between Identified Factors

Private Industry Pairwise Comparison

Factor	Very Important	Important	Moderately Important	Equal	Moderately Important	Important	Very Important	Factor
Participation in Existing Networks								Limited Knowledge of Forest Management
Participation in Existing Networks								Enhanced Job Opportunities for Women
Participation in Existing Networks								Absenteeism
Limited Knowledge of Forest Management								Enhanced Job Opportunities for Women
Limited Knowledge of Forest Management								Absenteeism
Enhanced Job Opportunities for Women								Absenteeism

Please indicate your stakeholder group.

- ☐ Landowner
- ☐ Non-Profits
- ☐ Forester (Federal or State Agency)
- ☐ Forester (Private)
- ☐ Other (Please specify): ____________________

Thank you for participating in the survey!
